# Seroprevalence of *Toxoplasma gondii* and Its Association with Sociodemographic Factors, Awareness and Exposure Practices Among Pregnant Women Attending Pumwani Maternity Hospital, Nairobi, Kenya

**DOI:** 10.3390/pathogens15090969

**Published:** 2026-09-11

**Authors:** Cherotich Jesca Tangus, Bruno Enagnon Lokonon, Ndichu Maingi, James Chege Nganga, Davis Karanja Njuguna, Kariuki Njaanake, Hellen Njeri Maingi, Esther Gwae Kimaro, Kennedy Kwasi Addo, Gloria Ivy Mensah, Bassirou Bonfoh

**Affiliations:** 1 Department of Veterinary Pathology, Microbiology and Parasitology, Faculty of Veterinary Medicine, University of Nairobi, Nairobi P.O. Box 29053-00625, Kenya; nmaingi@uonbi.ac.ke (N.M.); jchege@uonbi.ac.ke (J.C.N.); dnkaranja@uonbi.ac.ke (D.K.N.); hmaingi@uonbi.ac.ke (H.N.M.); 2 Laboratoire de Biomathématiques et d’Estimations Forestières (LABEF), Faculty of Agronomic Sciences (FSA), University of Abomey-Calavi, Abomey-Calavi P.O. Box 01 BP 2009, Benin; brunolokonon@gmail.com; 3 Centre Suisse de Recherches Scientifiques (CSRS), Abidjan P.O. Box 01 BP 1303, Côte d’Ivoire; bassirou.bonfoh@csrs.ci; 4 Department of Medical Microbiology, University of Nairobi, Nairobi P.O. Box 19676-00202, Kenya; kn@uonbi.ac.ke; 5 Nelson Mandela African Institution of Science and Technology, Arusha P.O. Box 447, Tanzania; esther.kimaro@nm-aist.ac.tz; 6 Noguchi Memorial Institute for Medical Research, University of Ghana, Legon, Accra P.O. Box LG-581, Ghana; kaddo@noguchi.ug.edu.gh (K.K.A.); gmensah@noguchi.ug.edu.gh (G.I.M.)

**Keywords:** toxoplasmosis, antenatal care, risk factors, maternal health, foodborne zoonosis, One Health, Kenya

## Abstract

**Background:** *Toxoplasma gondii* infection during pregnancy may lead to congenital toxoplasmosis and serious pregnancy complications. In Kenya, however, toxoplasmosis is not screened during routine antenatal care, and there is limited local data on maternal seroprevalence and associated risk factors. This study determined the seroprevalence of *T. gondii* infections. It examined its association with socio-demographic, obstetric characteristics, awareness levels, and exposure behaviours among pregnant women visiting the antenatal clinic at Pumwani Maternity Hospital in Nairobi, Kenya. **Methods:** A cross-sectional study was conducted among 298 pregnant women consecutively recruited at Pumwani Maternity Hospital in Nairobi County between July and October 2025. Data on socio-demographics, obstetric characteristics, knowledge, and exposure practices were collected using a structured questionnaire. Blood specimens were obtained from consenting prenatal patients, and anti-*T. gondii* IgG antibodies were detected by indirect enzyme-linked immunosorbent assay (ELISA). Associations between seropositivity and risk factors were assessed using univariable logistic regression, followed by two separate multivariable models. Variables with *p* < 0.25 were considered for multivariable analysis, followed by backward likelihood-ratio elimination. Model calibration was assessed using the Hosmer–Lemeshow test. **Results:** The overall seroprevalence of anti-*T. gondii* IgG was 24.5% (73 of 298). In the sociodemographic model, women who were self-employed (adjusted odds ratio [AOR] = 0.26, 95% CI: 0.13–0.53) and formally employed (AOR = 0.25, 95% CI: 0.11–0.59) had lower odds of seropositivity than unemployed women. Women aged 25–33 years had higher odds than those aged 18–24 years (AOR = 2.45, 95% CI: 1.06–5.62). Having two children was associated with lower odds than a first birth (AOR = 0.40, 95% CI: 0.18–0.90), while parity of four or more showed a borderline increase in odds (AOR = 3.11, 95% CI: 0.99–9.76). In the knowledge/exposure model, good awareness of population risk groups was linked to higher odds of seropositivity (AOR = 3.34, 95% CI: 1.21–9.24), but exposure-practice variables were not independently associated. Stratified analyses showed significant demographic differences in exposure practices, such as eating undercooked meat in the third trimester of pregnancy (*p* = 0.021) and unwashed vegetable consumption by pregnant women from Kamkunji Sub-County (*p* = 0.005). Parity of three increased infection risk (*p* = 0.007). **Conclusions:** About 25% of participants showed evidence of prior *T. gondii* exposure. Seropositivity correlated with specific sociodemographic and obstetric factors as well as awareness of groups at risk. These results endorse targeted antenatal education that emphasises practical food safety and environmental hygiene, aligned with a One Health approach. Further longitudinal and multicenter research, including IgM and IgG avidity testing, is needed to differentiate recent from past infections and to better understand risks associated with congenital toxoplasmosis.

## 1. Introduction

The obligate intracellular protozoan and zoonotic pathogen *Toxoplasma gondii* chronically infects approximately one-third of the world’s population [1,2]. In immunocompetent individuals, most infections are asymptomatic or resolve on their own. However, if a woman becomes infected during pregnancy, it can threaten the foetus through transplacental transfer, which the immature immune system cannot control [2]. Congenital toxoplasmosis can lead to miscarriage, stillbirth, or neurological and visual impairments [2,3]. Most severe cases of congenital toxoplasmosis involve macular chorioretinitis and hydrocephalus with intracranial calcifications [2,4]. The extent of foetal damage depends on the gestational age upon maternal infection, whereby early pregnancy infections cause severe outcomes but have lower transmission rates. In contrast, late pregnancy is associated with higher transmission but has less severe effects. The precise timing of maternal infection is important, even when asymptomatic, making laboratory testing necessary for early detection and prevention [2].

*Toxoplasma gondii* infection is a major maternal health concern worldwide, as evidenced by a recent review on pregnant women that reported a pooled IgG seroprevalence of 32.9% and an IgM seroprevalence of 1.9%, with higher rates in the WHO Eastern Mediterranean and African regions [5]. A review of African studies reported an IgG prevalence of 42.89% among women, with East Africa at 48.41%, the second-highest regional burden [6]. West Africa’s data match national estimates [6,7]. The high prevalence in sub-Saharan Africa is linked to environmental conditions such as warm, humid soil; a lack of safe water; poor sanitation; poor food hygiene; and the absence of routine prenatal screening in resource-constrained areas [6].

Human infection mainly occurs through ingesting tissue cysts from raw or undercooked meat or consuming oocysts from contaminated soil, water, fruits, vegetables, or food-contact surfaces. Other known risk factors include contact with cat faeces, especially from hunting or roaming cats; drinking untreated water or unpasteurized milk; and poor hand or utensil hygiene after handling raw meat, soil, or unwashed produce [8,9,10]. Since transmission involves humans, animals, food systems, and the environment, the One Health framework is ideal for guiding data collection and interventions [6,11].

In Kenya, *Toxoplasma gondii* infection impacts both immunocompetent and immunocompromised individuals, with seroprevalence differing by region and population group. National data show that approximately 35% of pre-school children, 60% of school-aged children, and 54% of HIV-positive adults are infected, with higher exposure in pastoral communities [12]. Studies in Nairobi report an anti-*T. gondii* IgG seroprevalence of 22–27% among pregnant women, while coastal Kenya shows much higher rates [13,14]. Similar regional variations are seen across sub-Saharan Africa, influenced by environmental factors, socioeconomic status, dietary habits, and contact with infection sources. Despite this substantial burden, Kenya’s maternal healthcare program has not yet incorporated routine antenatal screening for toxoplasmosis.

While previous studies have shown the presence of *T. gondii* antibodies among pregnant women in Nairobi, there is limited data from public maternity clinics that primarily serve low- and middle-income urban and peri-urban populations. Moreover, few Kenyan research efforts have examined how sociodemographic factors, awareness, and exposure behaviours together influence maternal *T. gondii* seropositivity within a One Health framework. This gap in knowledge hampers the development of targeted, locally tailored interventions to effectively lower the risk of maternal infection and congenital toxoplasmosis.

The rapid urbanisation in Nairobi County, along with the growth of informal settlements, evolving food systems, and close interactions among humans, animals, and the environment, may heighten the risk of *T. gondii* transmission. Pregnant women can be exposed through eating undercooked meat, unwashed produce, untreated water, contact with contaminated soil, unsafe food handling, and contact with cat faeces [8,9,10,12,13,14]. However, the specific impact of these behavioural, social, and environmental factors remains insufficiently studied among pregnant women attending public referral maternity hospitals in Nairobi.

This study was conducted at Pumwani Maternity Hospital, the largest public maternity hospital in Kenya, which mainly serves a low-income urban and peri-urban population from Nairobi County. While prior research has documented the seroprevalence of *Toxoplasma gondii* among pregnant women in Nairobi, data from this underserved group are scarce. The study sought to determine the prevalence of anti-*Toxoplasma gondii* IgG antibodies and explore their links to sociodemographic factors, awareness levels, and exposure practices within a One Health framework. By identifying key predictors of seropositivity and explaining demographic differences in awareness and behaviours, the findings provide context-specific evidence that can support targeted antenatal health education and risk reduction strategies in public maternal healthcare settings.

## 2. Materials and Methods

### 2.1. Study Area and Design

The study was carried out at Pumwani Maternity Hospital in Starehe Sub-County, Nairobi County, Kenya. As the country’s largest public maternity referral hospital, Pumwani serves pregnant women from both urban and peri-urban sub-counties within Nairobi County. The hospital admits patients from a range of socioeconomic backgrounds, including those from formal residential areas, informal settlements, and peri-urban communities. This broad catchment area provides access to a heterogeneous study population, making the facility well-suited to investigating demographic, behavioural, and environmental factors associated with *Toxoplasma gondii* exposure during pregnancy.

A hospital-based cross-sectional study was conducted over four months (July to October 2025). This design enabled simultaneous estimation of *Toxoplasma gondii* IgG seroprevalence and assessment of associations between seropositivity and participants’ sociodemographic, obstetric, knowledge, practice, and behavioural exposure profiles.

### 2.2. Study Population

#### 2.2.1. Inclusion Criteria

Prenatal patients attending the antenatal care (ANC) clinic at Pumwani Maternity Hospital during the study period were recruited if they were aged 18 years or older, lived in urban or peri-urban areas of Nairobi County or its immediate outskirts, provided written informed consent, and agreed to provide a venous blood sample (3 mL).

#### 2.2.2. Exclusion Criteria

Pregnant women with known or self-reported medical conditions or treatments that might weaken the immune system were excluded because such changes could impact antibody responses and affect the interpretation of anti-*Toxoplasma gondii* IgG serological results. Limiting participation to immunocompetent women created a more uniform group for assessing seroprevalence. Moreover, women who refused to participate or to provide a blood sample were also excluded.

Prenatal patients attending the antenatal care clinic at Pumwani Maternity Hospital during the study period were recruited using consecutive sampling. Recruitment took place after routine clinical consultations to minimise disruption to patient care.

### 2.3. Sample Size Estimation

The minimum sample size was estimated using the standard formula for a single-population proportion: *n* = Z^2·P·(1 − P)/L^2, where *n* represents the estimated sample size, Z = 1.96 (corresponding to a 95% confidence level), P is the expected seroprevalence, and L = 5% is the target margin of error (precision). This method was chosen because the number of pregnant women attending the antenatal clinic and meeting all eligibility criteria during recruitment couldn’t be accurately predicted during the study’s planning phase. The infinite-population formula is a widely accepted, conservative approach for calculating sample size in cross-sectional prevalence studies when exact population size is unknown.

Using a previously reported *Toxoplasma gondii* seroprevalence of 26.5% among pregnant women attending antenatal care at Kenyatta National Hospital, Nairobi [13], we calculated the minimum required sample size to be 299 participants. A total of 298 eligible pregnant women were enrolled, representing 99.7% of the target sample size. Although a finite population correction may be considered when the target population is known and relatively small, it was not applied because the accessible population could not be accurately defined during study planning and was expected to be substantially larger than the required sample size. The study was primarily powered to estimate seroprevalence and may therefore have had limited statistical power to detect weaker associations between seropositivity and individual risk factors.

### 2.4. Questionnaire Surveys and Interviews

A structured, pre-tested questionnaire created in English was used during face-to-face interviews by trained research assistants in either English or Kiswahili. The questionnaire was designed based on a review of relevant literature and expert advice to ensure content validity. It gathered data on sociodemographic details, obstetric history, awareness of toxoplasmosis, and exposure practices. Before the main study, it was pilot-tested with ten pregnant women at Pumwani Maternity Hospital to evaluate its clarity, understanding, and practicality. Feedback from this pilot helped refine the wording, order, and cultural relevance of the questions before the full study began. Data from the pilot were excluded from the final analysis. During data collection, completed questionnaires were reviewed daily to verify their completeness, consistency, and compliance with the study protocol.

#### 2.4.1. Knowledge Scoring

Knowledge of toxoplasmosis was assessed using three questions: whether participants had heard of it, their information sources, and whether they knew it could affect humans. Participants with 0 or 1 correct answer were classified as having low awareness; those with 2 or 3 correct answers were classified as having good awareness. Knowledge of toxoplasmosis transmission was assessed by asking about recognised sources of infection, including contaminated meat, water, unwashed vegetables, and soil. We also assessed awareness that pregnant women, infants, and immunocompromised individuals are particularly vulnerable to toxoplasmosis.

#### 2.4.2. Exposure Practices

Exposure practices were assessed by asking about behaviours before and during the current pregnancy. The questionnaire focused on consumption of undercooked meat, pork, or unboiled milk; eating raw or unwashed vegetables; contact with cat faeces; gardening without gloves; and other environmental exposures associated with *T. gondii* transmission.

Exposure history for both before and during pregnancy was collected, with a primary focus on exposures during the current pregnancy, as infection during this period can lead to congenital transmission and harm the foetus. Data were collected electronically using Open Data Kit on encrypted Android tablets and uploaded to the Afrique One ASPIRE server for secure storage.

### 2.5. Blood Sample Collection and Serological Analysis

A trained phlebotomist collected approximately 3 mL of blood specimen from each participant using sterile Vacutainer tubes. Each sample was labelled with a unique participant ID, kept in insulated cool boxes at 2 to 8 °C, and transported on the same day to the Kenya Institute of Primate Research (KIPRE) laboratory in Nairobi for analysis. KIPRE has the necessary facilities for serological testing and specimen storage. Blood specimens were processed in accordance with standard laboratory procedures and securely stored under appropriate conditions in accordance with the approved study protocol until analysis.

In the laboratory, blood specimens were centrifuged at 3200 rpm for 10 min to separate the serum. Clear, non-haemolytic serum was transferred into sterile, labelled cryovials and stored at −20 °C until analysis. The cold chain was maintained throughout transport and storage, and aliquoting minimised freeze–thaw cycles. During processing, haemolytic, lipaemic, or insufficient samples were identified, and additional samples were requested when possible. Anti-*Toxoplasma gondii* IgG antibodies were detected using the Vircell Toxoplasma ELISA IgG kit (VC G1027; Vircell, Granada, Spain), which has a sensitivity of 99% and a specificity of 98%, according to the manufacturer [15].

Samples were classified as negative if the index value was below 0.9, equivocal if between 0.9 and 1.1, and positive if above 1.1. Equivocal samples were retested in duplicate. If results remained equivocal after repeat testing, they were excluded from further analysis and reported as indeterminate. For quality assurance, samples were tested in duplicate, manufacturer-provided positive and negative controls were included on each plate, laboratory equipment was calibrated, and standard operating procedures were followed.

### 2.6. Ethical Considerations

This study was ethically approved by the Kenyatta National Hospital/University of Nairobi Ethics and Research Committee (P115/02/2025) and licensed by NACOSTI (reference NC50946). Recruited prenatal patients provided written informed consent after being informed of the study’s objectives, procedures, benefits, risks, and their right to cease participation at any time without penalty. Each participant was assigned a unique anonymised identification code, which was used to label questionnaires, blood samples, laboratory records, and electronic datasets. All study data were kept securely and were accessible only to authorised members of the research team.

### 2.7. Data Management and Statistical Analysis

Questionnaire and laboratory data were downloaded from the Afrique One ASPIRE server as .csv files, then cleaned, organised, and imported into Microsoft Excel. After merging with unique participant IDs, the dataset was imported into R for analysis [16]. All descriptive and multivariable analyses were conducted on the verified, merged dataset containing 298 participants with complete questionnaire and serological data, ensuring uniformity across analyses. Descriptive statistics summarised participant characteristics, awareness levels, exposure practices, and serological results. Seroprevalence was calculated as the proportion of ELISA-positive participants among those with definitive test outcomes, reported with 95% confidence intervals.

To account for the Vircell anti-*Toxoplasma gondii* IgG ELISA’s imperfect diagnostic performance—sensitivity of 99% and specificity of 98%—the true seroprevalence was calculated using the Rogan–Gladen estimator, TP = (AP + Sp − 1)/(Se + Sp − 1). Here, TP represents true prevalence, AP is the apparent prevalence, Se is the sensitivity, and Sp is the specificity. Both the apparent and adjusted prevalence estimates include their 95% confidence intervals.

Binary logistic regression was used to evaluate factors associated with anti-*Toxoplasma gondii* IgG seropositivity. Initially, univariable analyses were performed separately for (i) socio-demographic and obstetric variables, and (ii) knowledge and exposure-practice variables. Crude odds ratios (ORs), 95% confidence intervals (CIs), and *p* values were calculated.

Variables with a univariable likelihood-ratio *p* value < 0.25 were considered for inclusion in the multivariable model. Age was included in the socio-demographic model regardless of statistical significance due to its biological importance. Separate multivariable logistic regression models were developed for socio-demographic/obstetric variables and for knowledge/exposure-practice variables to prevent model redundancy and to evaluate the socio-demographic and behavioural factors independently. Final models were constructed using backward likelihood-ratio selection. Adjusted odds ratios (aORs) with 95% confidence intervals were reported, and model fit was checked with the Hosmer–Lemeshow goodness-of-fit test, where *p* ≥ 0.05 indicated good calibration.

## 3. Results

### 3.1. Socio-Demographic Characteristics of the Study Participants

The study involved 298 pregnant women with a median age of 29 years (IQR: 24–33) and a median parity of 2 (IQR: 1–3). The majority were residents of Embakasi (30.5%), followed by Kamukunji (15.8%), satellite peri-urban areas (13.8%), and Kasarani (11.4%) Sub-Counties. At enrollment, 18.5% of women were in the first trimester, 23.2% in the second, and 58.4% in the third. Most participants had completed secondary education (52.7%), with 36.2% having tertiary education and 11.1% primary education. Nearly half were self-employed (49.0%), while 27.5% were formally employed, and 23.5% were unemployed, as detailed in Table 1. Knowledge of toxoplasmosis was limited among participants: only 1.7% (5 women) fully understood it, 8.4% (25 women) knew the main transmission channels, and 5.4% (16 women) correctly identified high-risk groups.

Regarding dietary and behavioural practices, 5.4% (16 of 298) reported eating undercooked meat, and 83.6% (249 of 298) reported eating pork. Cat exposure was reported by 70.1% (209 of 298) of participants. Furthermore, 21.1% (63 of 298) consumed unboiled milk, and 41.3% (123 of 298) ate unwashed vegetables (Table 1).

### 3.2. Anti-Toxoplasma gondii IgG Seroprevalence

Among 298 pregnant women tested, 73 showed positive for anti-*T. gondii* IgG antibodies, resulting in an apparent seroprevalence of 24.5% (73/298; 95% CI: 19.6–29.4%). After adjusting for the Vircell ELISA IgG assay’s reported sensitivity (99%) and specificity (98%) using the Rogan–Gladen estimator, the estimated true seroprevalence was 23.2% (95% CI: 18.1–28.2%). The small difference between the apparent and adjusted figures suggests that the assay’s high diagnostic accuracy had little impact on the seroprevalence estimate. Participants with seropositivity were found in all major residential sub-counties within the study population. Although seroprevalence varied by location, these differences were not statistically significant (*p* = 0.440).

### 3.3. Univariable Associations Between Demographic Characteristics and Seropositivity

In the univariable analysis (Table 2), occupation (overall *p* = 0.001) and parity (overall *p* = 0.003) showed significant links to anti-*T. gondii* IgG seropositivity, while education (overall *p* = 0.188). Residence and pregnancy trimester did not have significant associations with seropositivity.

Compared to unemployed participants, pregnant women who are self-employed (OR = 0.32, 95% CI: 0.17–0.60; *p* < 0.001) and those who are employed (OR = 0.34, 95% CI: 0.16–0.72; *p* = 0.004) had significantly lower crude odds of being seropositive. Pregnant women with two children (parity two) also had reduced odds of seropositivity compared to primiparous women (OR = 0.44, 95% CI: 0.22–0.88; *p* = 0.020), while those with four or more children (parity ≥ 4) showed a borderline positive relationship (OR = 2.37, 95% CI: 0.99–5.70; *p* = 0.053). Higher levels of education were linked to a trend of decreasing crude odds of seropositivity, although this overall trend did not reach statistical significance.

### 3.4. Multivariable Demographic Predictors of Seropositivity

After adjusting for multiple variables, occupation, parity, and age remained in the final model (Table 3). Compared to unemployed participants, both self-employed (AOR = 0.26, 95% CI: 0.13–0.53; *p* < 0.001) and employed pregnant women (AOR = 0.25, 95% CI: 0.11–0.59; *p* = 0.001) were significantly less likely to test seropositive. Prenatal patients with two children (parity two) had notably lower odds of seropositivity than primiparous women (AOR = 0.40, 95% CI: 0.18–0.90; *p* = 0.027). Participants aged 25–33 years showed higher adjusted odds of seropositivity compared to those aged 18–24 years (AOR = 2.45, 95% CI: 1.06–5.62; *p* = 0.035). Although women with parity ≥ 4 exhibited higher adjusted odds (AOR = 3.11, 95% CI: 0.99–9.76), this result was borderline significant (*p* = 0.052). The model demonstrated good calibration, with Hosmer–Lemeshow *p* = 0.456.

### 3.5. Knowledge and Practice Variables Associated with Toxoplasma gondii Seropositivity

Among the variables related to knowledge and exposure-practice, awareness of population groups at risk for toxoplasmosis was significantly linked to anti-*T. gondii* IgG seropositivity (overall *p* = 0.023) (Table 4). Prenatal patients who had good knowledge about at-risk groups showed higher crude odds of seropositivity compared to those with poor knowledge (OR = 3.34, 95% CI: 1.21–9.24; *p* = 0.020).

Knowledge of transmission, specific risk-group items, undercooked meat consumption, pork risk, milk intake, and vegetable consumption all met the predefined screening criterion (*p* < 0.25) and were included in the multivariable analysis. Conversely, cat exposure showed no significant association with seropositivity (overall *p* = 0.351) and was therefore excluded from the multivariable model.

After using backward likelihood-ratio selection, only knowledge of at-risk population groups persisted in the final model (Table 5). Participants with good awareness of these groups had higher odds of anti-*T. gondii* IgG seropositivity compared to those with poor knowledge (AOR = 3.34, 95% CI: 1.21–9.24; *p* = 0.020). After adjustments, none of the exposure-practice variables were independently linked to seropositivity. The model demonstrated an adequate fit based on the Hosmer–Lemeshow test.

### 3.6. Stratified Associations Between Demographic Characteristics, Awareness, and Exposure Practices

Stratified analyses identified several significant associations between demographic characteristics, awareness, and exposure practices (Table 6). Third-trimester participants were more likely to report consumption of undercooked meat than those in the first or second trimester (*p* = 0.021). Consumption of unwashed vegetables was significantly associated with residence in Kamukunji Sub-County (*p* = 0.005) and satellite peri-urban areas (*p* = 0.019), as well as parity of three (*p* = 0.007).

Pork consumption was significantly associated with self-employment (*p* = 0.002), secondary education level (*p* = 0.014), and age 34–42 years (*p* = 0.017). Consumption of unboiled milk was significantly associated with age 16–24 years (*p* = 0.029).

Awareness of groups at risk of toxoplasmosis was significantly associated with first-trimester pregnancy (*p* = 0.001) and age 34–42 years (*p* = 0.010). Awareness of a second risk-group item was significantly associated with self-employment (*p* = 0.010).

## 4. Discussion

The prevalence of *Toxoplasma gondii* infection among pregnant women attending antenatal care at Pumwani Maternity Hospital in Nairobi, Kenya, was assessed, along with its association with sociodemographic characteristics, awareness, and exposure habits. Approximately 24.5% of participants tested positive for anti-*T. gondii* IgG, indicating that nearly one in four had prior exposure. After adjusting for confounding variables, unemployment and having four or more previous births were associated with higher infection rates, whereas awareness and individual exposure habits were not. Variations in awareness and practices across demographic groups indicate that broader socioeconomic and environmental elements may have a greater influence on exposure than individual behaviours.

Adjustment of seroprevalence estimates using the Rogan-Gladen estimator resulted in negligible changes, indicating high accuracy of the ELISA test employed. The observed seroprevalence implies ongoing exposure to *T. gondii* within this population, although the specific transmission pathways could not be determined due to the cross-sectional study design. Established sources of *T. gondii* include contaminated food, water, soil, and environmental reservoirs. Notably, approximately three-quarters of participants tested negative, signifying continued susceptibility to infection during pregnancy and the associated risks of congenital toxoplasmosis and related fetal complications [2,3].

The infection rate identified in this study is consistent with previous findings from Nairobi, which reported rates of 22% at Kenyatta National Hospital and 27% at Aga Khan University Hospital [13,14]. Comparable seroprevalence has also been documented among pregnant women in Dar es Salaam (27.2%), Uganda (16.4%), Mwanza (30.9%), and Nigeria (30.4–34.7%) [9,10,17,18]. Collectively, these data indicate that pregnant women attending urban clinics across sub-Saharan Africa experience similar levels of exposure to *T. gondii*, despite differences in geographic location and population characteristics. Their locations and backgrounds differ.

The seroprevalence observed in this study is lower than the average rates reported in recent African reviews (approximately 40% to 45%) and below the global average of 32.9% [5,6]. This difference may reflect differences in environmental conditions, dietary habits, sanitation, socioeconomic status, study populations, or methodological methods, rather than a true decline in transmission. Comparisons between studies should account for the diagnostic methods employed. The present study used the Vircell anti-*Toxoplasma gondii* IgG ELISA, which has also been applied in studies involving workers in Iran, veterinarians in Portugal, and patients with multiple sclerosis in Iran [19,20,21]. Even when identical assays are used, variations in reported seroprevalence are primarily attributable to differences in study populations, exposure risks, and local contexts. Consequently, seroprevalence findings should be interpreted in light of both the testing methodology and the characteristics of the study cohort.

Importantly, the result was anti-*T. gondii* IgG seropositivity, mainly indicating previous exposure rather than ongoing or recent infection. Therefore, the 24.5% figure should not be seen as the prevalence or incidence of active toxoplasmosis during pregnancy. Instead, it reflects the proportion of women with detectable IgG antibodies at the time of testing, offering an estimate of cumulative exposure in the study group.

About three-quarters of women in this group were IgG-seronegative, indicating no evidence of past *T. gondii* exposure at the time of testing. These women remain at risk of infection during pregnancy, a major public health concern given the risk of transmission to the baby and associated health problems. The presence of many seronegative women, alongside those already exposed, highlights a real opportunity for prevention during pregnancy. Measures such as cooking meat thoroughly, handling food safely, practising good hygiene around soil or animals, and using safe water can reduce the risk of infection during pregnancy [1,22].

These results place the Pumwani group within the wider context of *T. gondii* exposure among pregnant women and add local data from Nairobi. However, IgG seroprevalence alone does not reveal the main routes of exposure in this group; it is a starting point for examining behavioural, socioeconomic, and environmental factors. Using a One Health approach, education for pregnant women should go hand in hand with efforts to improve food safety, reduce risks from animals, reduce environmental contamination, and ensure access to safe water, since many interrelated factors may contribute to *T. gondii* exposure.

The study shows a link between employment status and anti-*Toxoplasma gondii* IgG seropositivity. Women who are self-employed or have formal jobs are less likely to test positive than those who are unemployed, even after considering age, education, and number of children. This suggests that socioeconomic status may affect past *T. gondii* exposure in this group [18]. Employment status probably does not directly cause exposure but instead reflects differences in resources and exposure patterns. Comparable associations have been reported elsewhere in sub-Saharan Africa; for instance, in Ghana, lower educational attainment was linked to increased infection odds [23]. Studies in Nigeria and northern Tanzania associated socioeconomic disadvantage with greater exposure and reduced awareness of toxoplasmosis [8,9,24]. However, this relationship is not universal. In Mwanza, Tanzania, employed women and business owners demonstrated higher infection odds than peasant farmers, indicating that the impact of socioeconomic status may vary according to local practices and conditions [18].

Because this study is cross-sectional, it cannot show whether employment status actually causes differences in seropositivity. Also, anti-*T. gondii* IgG seropositivity mostly shows past exposure, which could have happened before the current pregnancy or job. So, current employment might reflect long-term socioeconomic status instead of the exact situation when exposure happened. Other factors that were not measured, like household income, food sources, water and sanitation, housing, and job activities, could also affect the results [7].

The differences seen between socioeconomic groups highlight the need to address both broader social factors and personal prevention steps. Preventing toxoplasmosis during pregnancy requires better access to safe food, clean water, good sanitation, and trustworthy health information [5]. Long-term studies that track socioeconomic status, food sources and preparation, water and sanitation, job activities, and environmental exposures are needed to better understand how these factors relate to *T. gondii* exposure [25].

Age was independently associated with seropositivity, with women in the middle reproductive-age group having higher odds than the reference group. This may be because older women have had more time to be exposed to environmental, foodborne, or household sources of *T. gondii*. Similar trends have been observed among pregnant women in sub-Saharan Africa, where higher maternal age is linked to greater seroprevalence. This is biologically plausible, as *T. gondii* IgG typically reflects past exposure and can accumulate over time. However, this pattern was not observed across all age groups in our study, suggesting that age alone does not explain the risk of exposure in this group.

Parity showed a non-linear relationship in the analysis. Women with two previous births had lower odds of seropositivity, while those with four or more births had higher, but only borderline, odds. This matches earlier studies that found higher *T. gondii* seroprevalence in women with more births or reproductive experience [17,21]. One reason could be that having more children means more time exposed to household, dietary, and environmental risks. However, since there was no clear dose-response in this study, this explanation is limited. Differences between studies may be due to factors like maternal age, socioeconomic status, diet, environment, and when infection happened. So, parity should be seen as a sign of different exposure histories, not a direct cause of *T. gondii* infection.

Pregnant women who knew more about *T. gondii* risk groups were also more likely to be seropositive. This does not mean that knowledge increases the risk of exposure. It is possible that women learned about these risks after being exposed, perhaps through healthcare, community information, or personal experience. Another possibility is that women with more awareness were exposed through risk factors not fully covered by the questionnaire. Other studies in sub-Saharan Africa have also found that pregnant women often have limited knowledge of toxoplasmosis and that there are gaps between what they know and what they do to prevent infection [9,17,23,24]. Research on food safety among pregnant women shows that knowing about risks does not always lead to safer behaviours [24]. These results suggest that knowledge alone may not be enough to prevent exposure, especially when daily habits are shaped by environmental, dietary, and socioeconomic factors.

After adjustment, none of the assessed exposure-practice variables, such as eating undercooked meat or pork, drinking unboiled milk, or eating unwashed vegetables, was linked to seropositivity on its own. This is different from earlier studies, which found that eating undercooked meat, contaminated vegetables, and other foodborne exposures were important risk factors for *T. gondii* infection [8,9,24]. One reason for this difference may be that *T. gondii* exposure often builds up over time from many sources, not just from a single recent behaviour. Also, because the study relied on self-reported habits, there could be recall or social-desirability bias, and the IgG test shows past exposure that might have happened years before the current pregnancy. These timing issues may explain why current habits do not always match serological results. So, the lack of an adjusted association does not mean these practices are unimportant, but that their individual effects could not be shown in this group. In addition, the group with higher odds of seropositivity compared to the reference group may have had more time to be exposed to *T. gondii* from the environment, food, or household sources as they got older. Similar patterns have been seen in pregnant women in sub-Saharan Africa, where older mothers are more likely to be seropositive [8,21]. This makes sense biologically, since *T. gondii* IgG usually reflects exposure over a lifetime. However, this link was not seen in all age groups in this study, so age alone may not explain exposure in this population. The two previous births were associated with lower odds of seropositivity, whereas those with four or more births had higher, though only borderline, odds. This pattern aligns with findings from other studies reporting higher *T. gondii* seroprevalence among women with greater reproductive experience [17,21]. Increased parity may correspond to prolonged exposure to household, dietary, and environmental risks. However, the absence of a clear dose-response relationship in this study limits the strength of this explanation. Variations between studies may be attributable to differences in age, socioeconomic status, diet, environment, or timing of infection. Thus, while parity may help identify women with differing exposure histories, it should not be interpreted as a direct causal factor for *T. gondii* infection.

Eating unwashed vegetables was linked to lower seropositivity in the univariable analysis, but this link was not statistically significant and did not remain after adjustment. This result is surprising because raw or poorly washed vegetables can be contaminated with *T. gondii* oocysts from soil or water. Similar foodborne and environmental transmission routes have been reported in studies of toxoplasmosis among pregnant women. [8,9,24].

This result may be due to other participant factors, such as diet, where they live, socioeconomic status, or access to different foods. Also, current eating habits may not match behaviours at the time of infection. Studies from Kenya show that what mothers eat is influenced by economic conditions, food availability, and cultural preferences [26,27]. These factors make it hard to link specific eating habits to past *T. gondii* exposure. Since the association did not remain after adjustment, these results do not support the idea that eating unwashed vegetables is protective. Instead, they highlight the difficulty of connecting past *T. gondii* exposure to a single self-reported dietary habit in a cross-sectional study.

These findings have significant consequences for antenatal prevention. While raising awareness about toxoplasmosis remains important, health education should go beyond simply recognising the disease to include practical, context-specific actions that minimise exposure. These actions include thoroughly cooking meat, handling raw meat safely, washing fruits and vegetables, practising hand hygiene, using safe water, and maintaining proper environmental sanitation. Special focus should be placed on socioeconomically disadvantaged women and those with higher parity, though the evidence linking high parity to risk was less precise in the multivariable analysis. Since individual exposure behaviours were not independently linked to seropositivity after adjustment, interventions should avoid blaming personal behaviour alone. Instead, antenatal counselling should be personalised, considering the social, dietary, environmental, and reproductive factors that influence exposure. The lack of an independent link for individual practices also suggests that broader structural and environmental factors, not fully captured by the questionnaire, play a role. A One Health strategy integrating maternal health services with food safety, veterinary public health, animal health, water and sanitation, and environmental health efforts may offer a more holistic approach to reducing *T. gondii* exposure. Future multicenter and longitudinal research should assess these interventions and include diagnostic tools to differentiate past from recent infections, thereby better guiding prevention of congenital toxoplasmosis.

### Strengths and Limitations

One of the main strengths of this study is that it combined lab-confirmed anti-*Toxoplasma gondii* IgG testing with information on participants’ backgrounds, pregnancies, awareness, and exposure habits, all within a One Health approach. This allowed us to look at previous *T. gondii* exposure in relation to behaviours and circumstances among pregnant women at Pumwani Maternity Hospital, the largest public maternity hospital in Kenya. By including women from different urban and peri-urban areas of Nairobi, we gathered data from groups with a range of environmental and socioeconomic backgrounds, which helps make our findings more relevant to similar public antenatal care settings.

Several limitations must be considered when interpreting these results. First, as this was a hospital-based cross-sectional study, it cannot establish causality from the observed associations between participant characteristics and *T. gondii* IgG seropositivity. Additionally, since the study took place at a single public referral maternity hospital, the results may not apply to pregnant women in private clinics, smaller public health centers, or rural antenatal settings. While participants from various urban and peri-urban areas were included, the sample does not represent the full range of geographic and socioeconomic backgrounds of pregnant women across Kenya. 

Secondly, 298 participants were enrolled, just one short of our target of 299, representing 99.7% of the planned sample size. The main purpose of the sample size was to estimate *T. gondii* seroprevalence rather than to detect weaker links between seropositivity and specific factors. As a result, some associations that did not reach statistical significance could be due to limited statistical power, especially for variables with small subgroups or rare events.

Third, *T. gondii* exposure was assessed through anti-*T. gondii* IgG tests. A positive IgG result signifies past exposure but does not reveal when the infection occurred or whether it was before or during the current pregnancy. Since we did not include anti-*T. gondii* IgM or IgG avidity tests, we cannot estimate how many women had recent infections or directly determine the risk of fetal transmission. Consequently, our results should be viewed as evidence of previous exposure rather than current or recent toxoplasmosis.

Fourth, data on food habits, environmental contacts, animal exposures, and other behaviours were self-reported. This could introduce inaccuracies due to faulty memory or social desirability bias, potentially leading to under-reporting of risky behaviours and affecting the associations between exposures and seropositivity. Although we adjusted for certain sociodemographic and obstetric factors, confounding from unmeasured environmental, dietary, work-related, or behavioural factors may still exist.

Fifth, the small number of participants in certain residential sub-counties led to less accurate estimates and limited the ability to detect minor geographical differences in seropositivity. As a result, the lack of a clear link between residential sub-county and seropositivity should be interpreted with caution and does not rule out the possibility of localised spatial variations in exposure. Additionally, using residential sub-county as the geographical unit is somewhat broad and may not sufficiently capture household-level environmental and behavioral factors that affect *T. gondii* transmission.

Pregnant women with known or self-reported medical conditions or treatments that could weaken the immune system were excluded because these factors might affect antibody responses and the interpretation of anti-*Toxoplasma gondii* IgG serology. As a result, our findings mainly apply to pregnant women with normal immune function. While this exclusion was suitable for our study and test analysis, it limits how directly our results can be applied to pregnant women with compromised immunity, an important and vulnerable group in Kenya’s public maternity services.

The results have practical significance for antenatal and public health initiatives. The higher odds of seropositivity among women with multiple pregnancies and vulnerable socioeconomic groups emphasise the need for targeted toxoplasmosis education during antenatal visits. This education should cover practical prevention methods, such as thoroughly cooking meat, safely handling raw meat, washing fruits and vegetables, practising hand hygiene, using clean water sources, and maintaining environmental sanitation. These precautions are especially vital in settings where diet and environment may increase *T. gondii* transmission.

From a One Health perspective, prevention should go beyond changing individual behaviours. Working together, antenatal care providers, food safety officials, environmental health services, and veterinary public health programs can help address the ways *T. gondii* spreads between people, animals, and the environment. Adding education about toxoplasmosis that fits the local context into regular antenatal counselling could be a good first step. Wider surveillance and strategies to reduce risk could further improve prevention at the community level.

Future research should employ multicentre and, where feasible, longitudinal designs to enhance representativeness and establish temporal relationships between exposure and infection. Incorporating anti-*T. gondii* IgM, IgG avidity testing, and molecular diagnostics would enable more precise differentiation between past and potentially recent infection. Studies involving a range of public and private antenatal facilities, including rural populations, would further elucidate the epidemiology of toxoplasmosis in Kenya and help determine whether the associations observed in this study are consistent across different populations and healthcare settings.

## 5. Conclusions

Previous exposure to *Toxoplasma gondii* is common among pregnant women attending Pumwani Maternity Hospital, highlighting toxoplasmosis as a significant yet under-recognised concern in maternal health in Nairobi. The pattern of seropositivity indicates that exposure is influenced more by the broader social and environmental context, such as living conditions, food access, and healthcare, rather than solely individual behaviours. This underscores the need for a prevention strategy that goes beyond merely increasing awareness, combining practical antenatal counselling with interventions targeting food safety, environmental hygiene, safe water, and other potential exposure routes. Antenatal services serve as a key platform for identifying women who could benefit from targeted prevention and integrating toxoplasmosis education into routine maternal care. Due to the limitations of IgG-only testing, multicenter longitudinal studies that include markers of recent infection are essential to better understand the timing and factors involved in maternal infection and to bolster evidence for preventing congenital toxoplasmosis in Kenya.

## Figures and Tables

**Table 1:** Socio-demographic, obstetric, knowledge, and exposure-practice characteristics of pregnant women attending Pumwani Maternity Hospital, Nairobi County, Kenya (*n* = 298).

Characteristic	Category	*n* (298)	Frequency (%)
Social demographic characteristics			
Age group (years)	18_24	88	29.5
	25_33	147	49.3
	34_42	58	19.5
	43_plus	5	1.7
Median age 29 (25–33)			
Education	Primary	33	11.1
	Secondary	157	52.7
	Tertiary	108	36.2
Occupation	Employed	82	27.5
	Self-employed	146	49
	Unemployed	70	23.5
Obstetric characteristics			
Parity	1	96	32.2
	4	27	9.1
	2	110	36.9
	3	65	21.8
Pregnancy trimester	1	55	18.5
	2	69	23.1
	3	174	58.4
Knowledge of Toxoplasmosis			
Knowledge of toxoplasmosis	Good	5	1.7
	Low	293	98.3
Knowledge of transmission	Good	25	8.4
	Low	273	91.6
Knowledge of risk groups	Good	16	5.4
	Low	282	94.6
Knowledge of the risk-group item	Good	15	5.0
	Low	283	95
Behavioural practices			
Consumption of undercooked meat	Yes	16	5.4
	No	282	94.6
pork consumption	Yes	249	83.6
	No	49	16.4
Cat exposure	Yes	209	70.0
	No	89	29.9
Use/drink boiled milk	Boiled milk	235	78.9
Use/drink unboiled milk	Unboiled milk	63	21.1
Consumption of unwashed vegetables	Eat unwashed vegetables	123	41.3
Consumption of washed vegetables	Eat washed vegetables	175	58.7

**Table 2:** Univariable associations between socio-demographic and obstetric characteristics and anti-*Toxoplasma gondii* IgG seropositivity (*n* = 298).

Variable	Category	Crude OR (95% CI)	*p* Value	Overall *p* Value
Education level	Primary	Ref.	—	0.188
	Secondary	0.58 (0.26–1.28)	0.178	
	Tertiary	0.45 (0.19–1.05)	0.064	
Occupation	Unemployed	Ref.	—	0.001
	Self-employed	0.32 (0.17–0.60)	<0.001	
	Employed	0.34 (0.16–0.72)	0.004	
Residence	Embakasi	Ref.	—	0.436
	Dagoretti	2.70 (0.42–17.40)	0.295	
	Kamukunji	1.55 (0.68–3.53)	0.295	
	Kasarani	0.87 (0.31–2.41)	0.788	
	Langata	4.06 (0.75–21.79)	0.103	
	Mathare	1.91 (0.71–5.12)	0.199	
	Roysambu	0.72 (0.19–2.71)	0.622	
	Ruaraka	0.00 (0.00–∞)	0.988	
	Satellite areas	2.10 (0.92–4.80)	0.078	
	Starehe	1.45 (0.46–4.55)	0.526	
	Westlands	0.51 (0.06–4.32)	0.534	
Age group (years)	18–24	Ref.	—	0.707
	25–33	1.14 (0.61–2.13)	0.673	
	34–42	1.08 (0.50–2.36)	0.844	
	≥43	3.40 (0.45–25.69)	0.236	
Parity	1	Ref.	—	0.003
	2	0.44 (0.22–0.88)	0.020	
	3	0.91 (0.45–1.84)	0.783	
	≥4	2.37 (0.99–5.70)	0.053	
Pregnancy trimester	First	Ref.	—	0.277
	Second	0.67 (0.31–1.47)	0.321	
	Third	0.57 (0.29–1.12)	0.104	

**Table 3:** Multivariable logistic regression analysis of demographic and obstetric factors associated with anti-*Toxoplasma gondii* IgG seropositivity (*n* = 298).

Variable	Category	Adjusted OR (95% CI)	*p* Value
Occupation	Unemployed	Ref.	—
	Self-employed	0.26 (0.13–0.53)	<0.001
	Employed	0.25 (0.11–0.59)	0.001
Parity	1	Ref.	—
	2	0.40 (0.18–0.90)	0.027
	3	0.80 (0.33–1.93)	0.612
	≥4	3.11 (0.99–9.76)	0.052
Age group (years)	18–24	Ref.	—
	25–33	2.45 (1.06–5.62)	0.035
	34–42	1.17 (0.39–3.46)	0.779
	≥43	2.75 (0.28–26.65)	0.383

**Table 4:** Univariable associations between knowledge and practice variables and anti-*Toxoplasma gondii* IgG seropositivity (*n* = 298).

Variable	Category	Crude OR (95% CI)	*p* Value	Overall *p* Value
Knowledge of transmission	Low	Ref.	—	0.179
	Good	1.84 (0.77–4.36)	0.167	
Knowledge of risk groups	Low	Ref.	—	0.023
	Good	3.34 (1.21–9.24)	0.020	
Knowledge of risk-group item	Low	Ref.	—	0.174
	Good	2.15 (0.74–6.26)	0.161	
Consumption of undercooked meat	No	Ref.	—	0.235
	Yes	1.93 (0.67–5.49)	0.221	
Pork consumption risk	Low risk	Ref.	—	0.228
	Moderate risk	1.35 (0.60–3.01)	0.469	
	High risk	2.01 (0.84–4.80)	0.115	
Cat exposure	No cat	Ref.	—	0.351
	Cat present	0.76 (0.43–1.34)	0.347	
Milk consumption	Boiled milk	Ref.	—	0.247
	Unboiled milk	1.45 (0.78–2.69)	0.241	
Vegetable consumption	Washed vegetables	Ref.	—	0.091
	Unwashed vegetables	0.62 (0.36–1.09)	0.095	

**Table 5:** Multivariable logistic regression analysis of knowledge and exposure-practice variables associated with anti-*Toxoplasma gondii* IgG seropositivity (*n* = 298).

Variable	Category	Adjusted OR (95% CI)	*p* Value
Knowledge of risk groups	Low	Ref.	—
	Good	3.34 (1.21–9.24)	0.020

**Table 6:** Significant associations between demographic characteristics and toxoplasmosis-related awareness and exposure practices were identified through stratified analysis (*n* = 298).

Demographic Variable	Stratum	Knowledge and Practice Variables	N	*p*-Value
Pregnancy trimester	First	Knowledge of risk groups	55	<0.001
Occupation	Self-employed	Pork consumption	146	0.002
Residence	Kamukunji	Consumption of unwashed vegetables	47	0.005
Parity	Third	Unwashed vegetables	65	0.007
Occupation	Self-employed	Knowledge of the risk-group item	146	0.010
Age group	34–42	Knowledge of risk groups	58	0.010
Education	Secondary	Pork consumption	157	0.014
Age group	34–42	Pork consumption	58	0.017
Residence	Satellite	Consumption of unwashed vegetables	41	0.019
Pregnancy trimester	Third	Consumption of undercooked meat	174	0.021
Education	Secondary	Knowledge of risk groups	157	0.028
Age group	16_24	Consumption of unboiled milk	88	0.029
Occupation	Self-employed	Knowledge of risk groups	146	0.031
Parity	Fourth_and above	Pork consumption	27	0.049

## Data Availability

The data supporting the findings of this study are included within the manuscript.

## Abbreviations

The following abbreviations are used in this manuscript:

ELISA	Enzyme-Linked Immunosorbent Assay
KIPRE	Kenya Institute of Primate Research
OD	optical density
NACOSTI	the National Commission for Science, Technology and Innovation
IgG	Immunoglobulin G
*T. gondii*	*Toxoplasma gondii*
ANC	Antenatal care
AOR	Adjusted odds ratio
CI	Confidence interval
